# Biofilms as Battlefield Armor for Bacteria against Antibiotics: Challenges and Combating Strategies

**DOI:** 10.3390/microorganisms11102595

**Published:** 2023-10-20

**Authors:** Sara Bano, Noor Hassan, Muhammad Rafiq, Farwa Hassan, Maliha Rehman, Naveed Iqbal, Hazrat Ali, Fariha Hasan, Ying-Qian Kang

**Affiliations:** 1 Applied Environmental and Geomicrobiology Laboratory, Department of Microbiology, Quaid-i-Azam University, Islamabad 45320, Pakistan; 2 Industrial Biotechnology Division, National Institute for Biotechnology and Genetic Engineering-College, Pakistan Institute of Engineering and Applied Sciences, Islamabad 44000, Pakistan; 3 Department of Microbiology, Balochistan University of Information Technology, Engineering and Management Sciences, Quetta 87300, Pakistan; 4 Department of Biotechnology & Informatics, Balochistan University of Information Technology, Engineering and Management Sciences, Quetta 87300, Pakistan; 5 The Department of Paediatrics and Child Health, Aga Khan University, Karachi 74800, Pakistan; 6 Key Laboratory of Environmental Pollution Monitoring and Disease Control, Ministry of Education of Guizhou, Guiyang 550025, China; 7 Key Laboratory of Medical Microbiology and Parasitology, School of Basic Medical Sciences, Guizhou Medical University, Guiyang 550025, China

**Keywords:** antibiotic resistance, biofilms, CRISPR/Cas, eDNA, exopolysaccharides, nanoparticle, quorum sensing, phages

## Abstract

Bacterial biofilms are formed by communities, which are encased in a matrix of extracellular polymeric substances (EPS). Notably, bacteria in biofilms display a set of ‘emergent properties’ that vary considerably from free-living bacterial cells. Biofilms help bacteria to survive under multiple stressful conditions such as providing immunity against antibiotics. Apart from the provision of multi-layered defense for enabling poor antibiotic absorption and adaptive persistor cells, biofilms utilize their extracellular components, e.g., extracellular DNA (eDNA), chemical-like catalase, various genes and their regulators to combat antibiotics. The response of biofilms depends on the type of antibiotic that comes into contact with biofilms. For example, excessive production of eDNA exerts resistance against cell wall and DNA targeting antibiotics and the release of antagonist chemicals neutralizes cell membrane inhibitors, whereas the induction of protein and folic acid antibiotics inside cells is lowered by mutating genes and their regulators. Here, we review the current state of knowledge of biofilm-based resistance to various antibiotic classes in bacteria and genes responsible for biofilm development, and the key role of quorum sensing in developing biofilms and antibiotic resistance is also discussed. In this review, we also highlight new and modified techniques such as CRISPR/Cas, nanotechnology and bacteriophage therapy. These technologies might be useful to eliminate pathogens residing in biofilms by combating biofilm-induced antibiotic resistance and making this world free of antibiotic resistance.

## 1. Introduction

Biofilms are defined as immobile microbial (e.g., bacteria) communities, which have an innate ability to grow and colonize on various surfaces including medical implants, catheters, and sutures [1]. Bacteria are either live in free-living planktonic mode or attached to the surface within biofilms, enclosed by a polymeric matrix. Therefore, bacteria exhibit two modes of growth: the free-living planktonic mode or the sessile, surface-attached mode within biofilms, which are structured communities encased in a self-produced polymeric matrix [2,3]. The forming ability of biofilms provides a dominant mode of growth for bacteria in nature [4]. Biofilms provide complex systems comprised of many species and possessing high cell densities ranging from 10^8^ to 10^11^ cells g^–1^ wet weight [5]. Biofilms can self-produce extracellular polymeric substances, which contribute to intensive infections, leading to extensive and expensive treatments [6]. The architecture of biofilms comprises microbial aggregate surrounded by an extracellular matrix, consisting of various polymers like exopolysaccharides (EPS), proteins, eDNA, and other amyloidogenic proteins [7]. The cells in multilayered biofilms are arranged closely with each other, either in contact with the surface, e.g., with substratum, or in flocs, which constituent a mobile form without making contact with the substratum [3].

Based on matrix properties and intercellular interactions, e.g., social and physical contact, biofilms present a different lifestyle to bacteria than free-living bacterial cells. Therefore, bacterial communities within biofilms possess new emergent properties, which are not present in free-living bacterial cells [8]. Furthermore, the cells within biofilms undergo differentiation due to multiple factors, e.g., local conditions such as pH, and, most importantly, due to molecular factors, e.g., the expression of specific genes and proteins required for the growth and development of bacteria in spatially heterogeneous ecosystems, which provides another source of heterogeneity to bacterial communities [9]. The biofilm-based emergent properties include new structures, functions and activities, new patterns and novel properties that arise during and after biofilm development [10]. These emergent properties, such as physical and social contact among the microbial communities, increased antibiotic resistance and an enhanced rate of gene exchange, are governed by the surrounding EPS matrix, which encloses bacterial cells within biofilms and is mostly composed of eDNA, lipids, proteins and sugars [11].

Biofilms cause approximately 80% of chronic and reoccurring infections in humans [7]. In the USA, biofilms act as the etiologic agent for around 60% of all chronic infections [12,13]. According to Omar et al. [14,15], 1.96 million cases of biofilm-based infections are reported annually in the USA, causing 268,000 deaths and costing approximately USD 18 billion in direct treatment of such infections. Individuals with medical implants and medical devices as well as immunocompromised immunity are at high risk of biofilm-related infections [13]. *Mycobacterium tuberculosis* was observed to cause infections in patients carrying clinical biomaterials and prosthetic joints [14]. Similarly, *Streptococcus pneumoniae* and *Haemophilus influenza* are involved in chronic otitis media [15]. Treating these infections is very difficult as biofilms protect pathogens by making them resistant to a variety of antibiotics. Therefore, new alternative options to antibiotics are required for combating antibiotic-resistant biofilm bacterial communities, which include CRISPR/Cas, nanotechnology, bacteriophage therapy, etc. This review presents the current state of knowledge of biofilm-based antibiotic resistance in bacteria. Genes responsible for biofilm development and their potential against various classes of antibiotics as well as the key role of quorum sensing (QS) in developing biofilms and antibiotics resistance are also discussed in this review. Furthermore, this review also highlights new and modified techniques like clustered regularly interspaced short palindromic repeat (CRISPR)/CRISPR-associated (Cas) proteins, nanotechnology and bacteriophage therapy, which may be useful to combat biofilm-induced antibiotic resistance.

## 2. Biofilm Development and Molecular Biology

**Figure 1:**
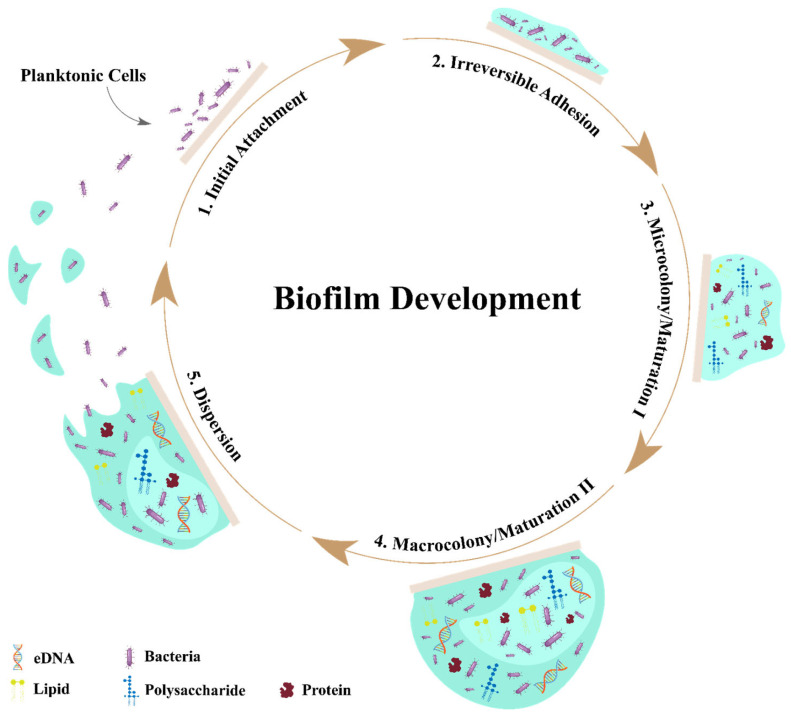
This figure represents the multiple-step process for biofilm formation in bacteria. Biofilm starts with bacterial attachment to a biotic or abiotic surface, which is irreversible, and then it matures through the replication of bacteria and production of EPS, ultimately leading to the dispersion of biofilms.

Bacteria start to develop biofilms under unfriendly conditions. In unfavorable conditions, microorganisms control the declaration of the progression of biofilm-shaping qualities through QS, nucleotide second courier-based flagging, and so on, which supply microorganisms with the ability to survive in unhabitual conditions such as UV radiation, extreme temperature, exposure to antibiotics and pH, high salinity, high pressing factor, limited nutrients, anti-infection agents, and so forth [16]. The development of biofilms is a multistep process that is initiated through the reversible attachment of bacteria on the surface (which could depend upon the protein), where bacteria are still vulnerable to antibiotics at this stage (Figure 1) [17]. The next step they follow is the replication of bacteria attached to the surface forming microcolonies and proceeding with the production of an extracellular polymer matrix around them. This process is completed within just a few hours after the attachment of bacteria to the surface [17]. At that stage, the biofilms grow in thickness and are practically visible, showing the maximum tolerance to antibiotics. Important properties of biofilm-growing bacteria are different from those of planktonic bacteria, and this has significant diagnostic and therapeutic consequences [18]. However, the development of biofilms solely depends on the type of gene, which each expresses differently to different types of antibiotics.

The genetics and environmental signals contribute to the regulation of biofilm development and dispersion in bacteria [19]. Three main players’ quorum sensing (QS), bis-(3′-5′)- cyclic diguanosine monophosphate (c-di-GMP), and small RNAs (sRNAs) are considered to be involved in the regulation of biofilm development and dispersion [20,21]. The QS is a special language used by bacteria for intercellular communication, which functions by small signal molecules called autoinducers [19]. Different genes control the QS pathway, comprising approximately 10% of the bacterial genome [22]. The QS pathway is required for the development and dispersal of biofilms, importantly considered as main regulators of biofilm dispersal [23,24].

Furthermore, c-di-GMP is a complex signaling network, considered to be a decider between the planktonic and biofilm-associated lifestyle of bacteria [25,26]. The c-di-GMP-based system regulates EPS synthesis, eDNA secretion, syntheses of pili and adhesins (a virulence factor) and controls cell death and motility [19]. Finally, sRNAs participate in a wide range of post-transcriptional gene regulation in bacteria [21,27,28]. Hence, sRNAs are considered to be involved in regulating the biofilm life cycle of bacteria, e.g., regulation of EPS synthesis, regulation of flagella, curli and cell surface structures as well as the regulation of biofilm-associated transcriptional and post-transcriptional regulators [21].

In addition, based on genomic analysis of bacteria, various genes are reported to be responsible for biofilm development and dispersion (Table 1). The *ndvB* gene was discovered in the genomic makeup of *Pseudomonas aeruginosa*, which encodes for glucosyltransferase that upregulates the synthesis of cyclic-b-(1,3)-glucans (important for biofilm formation) [29]. Moreover, *Escherichia coli* contains *HlyB–HlyD–TolC* complex, which is responsible for the exportation of hemolysin through biofilms, contributing to multi-drug resistance [30]. *RapA* genes, on the other hand, were also found to be responsible for biofilm-mediated resistance to penicillin in *E. coli* [31]. In *Vibrio cholera*, *tssC1* from the first type VI secretion system (T6S) was characterized as a virulent gene for toxin delivery in biofilm-related drug resistance [32]. Furthermore, *icaABCD* gene clusters were found to be important for enhancing virulence factors as well as for biofilm development and dispersion in methicillin-resistant *Staphylococcus aureus* [33]. Similarly, *icaA* and *icaD* genes were reported to be associated with the formation of slime and biofilm in *S. epidermidis* [34,35]. However, different types of genes are expressed in different types of bacteria, but the research community has consensus over the fact that biofilms contribute significantly to antibiotic resistance, thus creating severe medical complications and consequences.

## 3. Biofilm-Based Medical Problems

**Figure 2:**
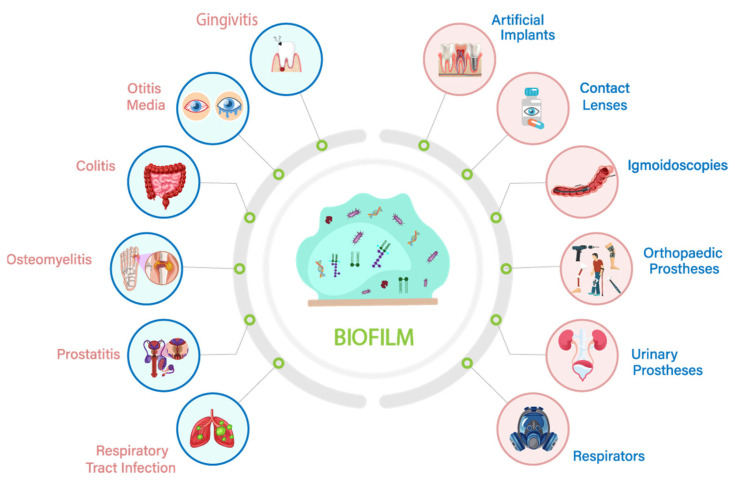
Pathogenic bacteria form biofilms on various artificial implants/devices, which leads to the development of various serious medical implications in humans.

Bacterial biofilms pose serious health issues due to their capabilities to combat external stressors, host defense systems and resist antibiotics; hence, they contribute to chronic infections [39]. Bacterial biofilms contribute to approximately 80% of chronic and recurrent microbial infections in the human body [7]. A large number of diseases are associated with bacterial biofilms in humans such as chronic osteomyelitis, chronic otitis media, chronic prostatitis, colitis, conjunctivitis, otitis, urethritis and vaginitis, a very short list of common diseases [40] (Figure 2). Biofilms also contribute to the development of gingivitis and infections in the oral cavity [23]. Furthermore, biofilms have been reported to infect artificial implants, contact lenses, orthopedic prostheses, respirators, sigmoidoscopies, urinary prostheses and ventricular assist devices [7]. Biofilms can even infect breast implants, leading to severe health issues. According to Davies [19], two main characteristics of biofilms contribute to problems associated with human biofilm infections. Firstly, biofilms are extremely resistant to immunological-based elimination as well as antimicrobial-agent-based killing and clearance. Secondly, biofilms provide shelter to individual bacterial cells inside the human body, making them invisible in surrounding tissues and the circulatory system, which might later cause acute illness as they are protected from antimicrobial agents even after vigorous use.

The distinguishing ability of bacteria to produce biofilms empowered them to become more pathogenic than ever. In particular, *S. Aureus* can colonize on medical devices and produce biofilms, which harbor teichoic acids, various genes, and eDNA in the EPS matrix, thus contributing to many nosocomial infections, e.g., pneumonia, bloodstream infections, endocarditis and osteomyelitis [41]. Moreover, the biofilm-producing abilities of *Klebsiella pneumonia* and *E. coli* make them cause urinary tract infections (UTIs) and other diseases [42]. *P. aeruginosa* is associated with chronic lung disease and cystic fibrosis due to its ability to form antibiotic-resistant biofilm in nosocomial settings. Moreover, these biofilms not only tolerate antibiotic treatment but also contribute to the genetic determinants causing mutations [43]. The infection of gums via the infiltration of the soft tissues and bones, a condition called periodontitis, is the outcome of *P. aerobicus* and *Fusobacterium nucleatum* biofilms [44]. The matrix of biofilm not only provides protection to bacteria from nutrient scarcity but also shear mechanical forces and altered pH [45], as well as blocking the access of antibiotics to bacteria residing in the matrix of biofilms. Hence, the biofilm matrix has the ability to provide additional resistance against antibiotics, which leads to the emergence of multi-drug-resistant bacteria [46].

## 4. Biofilm-Based Antibiotic Resistance

**Figure 3:**
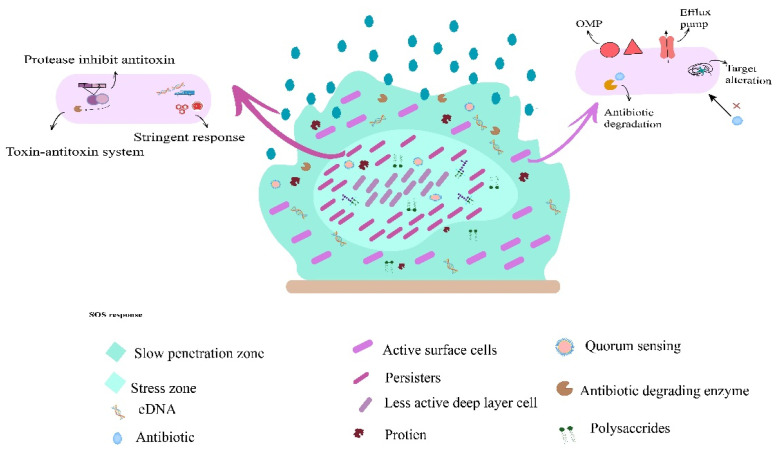
This figure represents the general hypothesized biofilm-mediated resistance mechanism in pathogenic bacteria.

Biofilms protect bacteria against multiple extreme factors, e.g., altered pH, osmolarity, nutrient scarcity, and mechanical and shear forces [45]. Most importantly, biofilms block the access of antibiotics to bacterial communities residing inside biofilms [47]. The resistance to antibiotics in biofilm communities occurs through multiple strategies, as explained in Figure 3, which include chemical alteration in the microenvironment in biofilms, slow or incomplete penetration through biofilm by antibiotics, and various subpopulations of microorganisms inside biofilms [3]. The multicellular consortia residing in biofilms form the basis of these mechanisms, which elevates the ability of bacteria to develop antibiotic resistance [48]. EPS plays an important role in the development of multicellular consortia by holding bacterial cells together, formulating a heterogeneous environment and enabling biofilms to work in a multicellular system [49]. Bacteria residing inside the biofilms showed a higher frequency of mutations and horizontal gene transfer as compared to bacteria living in planktonic states. The ability of bacteria to produce antibiotic-degrading enzymes inside EPS empowers them to mutate and resist antibiotics [50].

Generally, the nature of bacterial biofilm is a major factor in the development of multidrug resistance. The composition of biofilm mainly constitutes water, polysaccharides, or glycoprotein gel, which decreases the effective diffusion of antibiotics inside biofilms. The average rate of diffusion coefficients in biofilms is around 40% of the respective diffusion coefficient in pure water [47]. The presence of EPS and increased glycol protein inside the extracellular matrix of biofilm decreases the diffusion coefficient, which reduces the mobility of antibiotics inside biofilms. In addition to the physical immobility of antibiotics, the antibiotics inactivate or sequester by binding as they penetrate through the biofilm matrix. Briefly, this review aims to present the current state of knowledge about the biofilm defense mechanisms of different species of bacteria against different classes of antibiotics.

### 4.1. Cell Wall Targeting Antibiotics

The bacterial cell wall is one of the most important constituents for their survival and metabolism; hence, antibiotics are specifically designed or obtained to hinder bacterial cell synthesis and proliferation [51]. The D-alanyl-alanine part of peptidoglycan in the bacterial cell wall is cross-linked by residues of glycine, which is targeted by the β-lactams, thus inhibiting bacterial cell synthesis [52]. β-lactam antibiotics with their broad-spectrum range are effective against bacterial infections, caused by various pathogenic bacteria. This group of antibiotics, e.g., cephalosporin, is used to treat sinus, ear and urinary tract infections as well as to streptococcal and staphylococcal infections [53]. β-lactams and vancomycin antibiotics are among these agents, which were once considered the most effective antibacterials but have now been threatened by multidrug-resistant bacteria [54]. Particularly, biofilm-based resistance threatens the efficiency of β-lactam antibiotics.

The extracellular matrix of biofilms contains eDNA, which protects the bacteria against positively charged antibiotics [55] and escalates while interacting with some antibiotics. As observed in the case of *S. epidermidis* biofilms, eDNA doubles in concentration when interacting with vancomycin activity [56]. The increased amount of eDNA is responsible for chelating cations and activating signaling pathways of antimicrobial resistance like *PmrAB* and *PhoPQ* [57], causing the rapid spread of antibiotic resistance genes in bacteria [58]. Furthermore, sigma B factor in *S. aureus* controls some protein productions involved in developing resistance against cell wall active antibiotics like vancomycin [59]. Additionally, QS receptors, e.g., TraP quorum-sensing receptor, play an important role in *S. aureus* by showing elevated resistance against the cephalosporins. These receptors are associated with increased peptidoglycan synthesis and eDNA content of biofilms [60]. However, further extensive studies are required to access other QS receptors to analyze tolerance against the antibiotics [48].

### 4.2. Protein Synthesis Targeting Antibiotics

Protein synthesis is important for the conformational alignment of bacterial metabolism, which in turn provides an important target for antibiotics [61]. These antibiotics specifically target the formation of 30S initiation complex or the formation of 70S ribosome and, thus, prevent the elongation process of the polypeptide chain [62]. Aminoglycoside antibiotics have a specific affinity towards the 30S ribosomal subunit by altering the synthesis process, e.g., streptomycin and tobramycin bind with 30S initiation complex and block the formation of larger 70S subunits [63]. Moreover, doxycycline can hinder the binding of aminoacyl-tRNA by blocking the aminoacyl site (A site) of 30S ribosome [64]. The main role of aminoglycosides is to treat Gram-negative bacterial infections, particularly for the treatment of sepsis. Moreover, they are potent antibiotics for the treatment of tuberculosis, as streptomycin is among the few effective drugs against tuberculosis [65]. However, the biofilm-producing ability of pathogenic bacteria changed the scenario for protein synthesis targeting antibiotics by developing resistance in targeted pathogenic bacteria.

Recent studies implicated biofilm-based resistance in *P. aeruginosa* against tobramycin through periplasmic glucans (specifically expressed in biofilms), which bind to tobramycin and restrict their passage towards target proteins [66]. Moreover, two important factors, e.g., accessory gene regulator (agr) and sigma factor B (SigB), have been reported in *S. aureus* [67]. The agr is required by biofilms for colonization during the dissemination phase of infection, whereas alternative Sig B is needed for host tissue colonization during stress responses like antibiotic exposure, adhesion and expression [67]. Studies observed that Sig B also plays a major role in the upregulation of cell-surface proteins including fibronectin-binding protein A (FnBPA) and the downregulation of various exotoxins [68]. Consequently, findings stated that sigma B induces the production of biofilm through the up-regulation of the FnBPA gene upon exposure to aminoglycoside antibiotics [68]. Similarly, Sig-B-based antibiotic resistance patterns have been analyzed in clinically relevant pathogens after exposure to the glycopeptide class of antibiotics [69].

### 4.3. DNA Targeting Antibiotics

The antibiotic class of quinolones and fluoroquinolones inhibits bacterial type II topoisomerases and interferes in DNA coiling. Quinolones bind directly to the active site of topoisomerases, whereas fluoroquinolones stabilize enzyme–DNA complex to deactivate or interrupt the relegation step [70]. During nosocomial and genitourinary infections, fluoroquinolones and quinolones are prescribed for treatment [71]. Additionally, they are the first-line therapy for acute bacterial prostatitis and pyelonephritis and are recommended once other classes of antibiotics have failed to comply [72].

In recent years, researchers have reported that upon exposure to fluoroquinolone antibiotics, relaxing of supercoiling of DNA induced the formation of biofilms, particularly under aerobic conditions [73]. The strains, which were resistant to fluoroquinolone, were invasive and posed more threat to be transmitted among humans. The relaxation of DNA supercoiling triggered an increased amount of EPS production and eDNA, which restricted the induction of fluoroquinolone inside the biofilm matrix [57]. Later on, wild-type strains of *Campylobacter jejuni* and fluoroquinolone-resistant strains were injected into *Galleria mellonella* larvae, revealing that even a small amount of fluoroquinolone-resistant strain was responsible for the death of the tested animal as compared to wild-type strain [74]. Hence, antibiotic-resistant strain poses more pathogenic capabilities than wild type by the relaxation of supercoiled DNA.

### 4.4. Cell Membranes Targeting Antibiotics

Disturbing the plasma membrane initiates a cascade of events including depolarization of proteins, DNA and RNA, leading to bacterial cell death [75]. Many antibiotics work on this principle, among which macrolides like daptomycin and other cyclic lipopeptides are common. The daptomycin enters the phospholipid bilayer membrane and causes the loss of membrane potential, hence redirecting various important proteins for cell replication and division [76]. Daptomycin creates stress inside the cell membrane, leading to the activation of cytotoxic reactive oxygen species (ROS) by increasing peroxide production, thus neutralizing bacterial cells [77] *S. aureus* bacteremia and *S. aureus* endocarditis are treated using daptomycin in various regions of the world. Daptomycin has the ability to bind with pulmonary surfactants, so this antibiotic is not recommended for pneumonia [78]. However, most importantly, biofilms make daptomycin ineffective against pathogens, e.g., *S. aureus*.

The activity of daptomycin was low against bacteria producing adequate biofilms [79]. Parra-Ruiz et al. [80] reported that daptomycin was effective against planktonic *S. aureus* but showed ineffectiveness against biofilm-embedded *S. aureus*. In another study, biofilms producing *S. aureus* appeared to be unaffected by daptomycin due to the presence of a substance called catalase, which acted as an antagonist against cytotoxic peroxide and inhibited the production of ROS [81]. This study revealed the presence of catalase in biofilm-producing *S. Aureus*, which protected cells from daptomycin ROS capability [81]. The presence of a catalase survival mechanism against ROS activity underlines the importance of biofilm against environmental stressors.

### 4.5. Folic Acid Synthesis Targeting Antibiotics

Folic acid constitutes a necessary nutrient for the synthesis of protein and nucleic acid in bacteria, via the substrate para-amino-benzoic acid (PABA) [82]. The sulfonamide class of antibiotics are in particular used against the production of folic acids, acting as various competitive inhibitors of PABA and dihydropteroate synthetase, important enzymes for folic acid metabolism [83]. The sulphonamide drugs are recommended to treat thyroiditis, inflammation, and glaucoma inflammatory diseases and coughs [84]. They are even recommended to treat livestock diseases such as gastrointestinal and respiratory tract infections [84]. They are widely used with broad spectrum because they contain other moieties than a typical antibiotic like thiazide diuretics, acetazolamide and some COX-2 inhibitors [85]. However, biofilm-based resistance threatens the efficiency of sulfonamide antibiotics.

Integrons (genetic elements) play an important role in biofilm-based resistance to sulfonamide by spreading resistance genes among bacterial communities [86]. For example, *sul* genes inside the class 1 integrons encoded for sulfonamides resistance, e.g., sulfamethoxazole [87]. Biofilms are predominant in stress environments, which play an important role in dynamic exchange between attached and planktonic populations along with gene exchanges; among them, integrons play a major role in transferring antibiotic resistance genes [88]. The presence of class 1 integrons and the *sul* gene in water biofilms were investigated by Farkas et al. [86], where they identified that *S. Vitulinus, S. Saccharolyticus*, and *Enterococcus faecalis* contain class 1 integrons associated with biofilm formation. They emphasized the risk of bacterial resistance, which might be perpetuated through environmental species in the form of biofilms [86]. Moreover, *sul* genes restrict the absorption of sulfonamides inside bacterial cells, thus restricting the drug from reaching the target [89,90]. Antunes et al. [89] reported *sul* genes (*sul*1, *sul*2 and *sul*3) in 50 uropathogenic *E. coli* strains, which were resistant to sulfamethoxazole.

## 5. Quorum Sensing and Biofilm-Based Resistance

The QS plays a significant role in regulating biofilm development in both Gram-positive and Gram-negative bacteria [91]. The Gram-negative bacteria use acyl homoserine lactones (AHL) containing N-3-oxohexanoyl-l-homoserine lactone (3OC6-HSL) ring as a signal molecule to regulate the QS system [92,93], while oligopeptides are responsible for regulating the QS process in Gram-positive bacteria [94]. A Gram-negative bacterium, *P. aeruginosa*, uses two signaling systems of QS, rhlI/rhlI and lasl/lasR, which activate transcriptional regulators responsible for the synthesis of alginates, EPS, and toxic factors, thus leading to biofilm development [93]. Similarly, two-component sensing proteins are responsible for recognizing and regulating gene expression involved in biofilm formation in Gram-positive bacteria [94]. Although studies on the contribution of QS toward biofilm-based resistance against antibiotics are limited, it is believed to be a key player in the phenomenon of biofilm-based antibiotic resistance.

Previous studies highlight the role of QS in biofilm formation and subsequent development of resistance in pathogenic bacteria to antibiotics. In two different studies, biofilms developed by mutants of *P. aeruginosa*, lacking QS systems in biofilms, were more susceptible to tobramycin [95] and colistin [96] than wild-type biofilms. *E. faecalis*, possessing fsr QS system and gelE protease (responsible for the fratricidal release of eDNA in developed biofilms), was resistant to gentamicin, daptomycin and linezolid [97]. Additionally, QS receptors like AgrA and AgrC played an important role in *S. aureus*, indicating elevated resistance against cephalosporins, and further extensive studies are required to access other QS receptors to analyze tolerance against antibiotics [97]. The receptors of QS played an important role in increasing the EPS content of the biofilm, which in turn decreed the access of certain antibiotics like cephalosporins to the extracellular matrix.

Although QS contributes to the development of antibiotic resistance in bacteria, this system is now considered an attractive site for therapy by targeting through abiotic (e.g., altering pH) and biotic (e.g., enzymes) factors [98]. These factors have the ability to interfere with QS by degrading the signals required for biofilm development, a process known as quorum quenching (QQ) [99]. For example, two enzymes, lactonases and acylases, have been reported with the ability to degrade AHL [98]. In these two enzymes, acylases convert AHL to fatty acid and homoserine lactone by cleaving the AHL amide bond [100], while lactonases degrade the HSL ring of an AHL and generate acyl homoserines [99]. These enzymes can be acquired from natural biological sources such as lactonases (the paraoxonase (PON) family of enzymes), which are present in humans [101] and Drosophila [101]. It is believed that the enzymes are involved in manipulating microbial biofilms by interfering the microbial social interactions.

Furthermore, the QQ can be utilized as a method to assess the exact contributions of QS to biofilm resistance and tolerance [102]. Small molecules are usually used to increase biofilm susceptibility to antimicrobials in the QQ method. Brackman et al. [103] reported lowered biofilm resistance of *P. aeruginosa* and *Burkholderia cepacia* to tobramycin while using QS inhibitors cinnamaldehyde and baicalin hydrate. By inhibiting receptors of TraP quorum-sensing in *S. aureus* using hamamelitannin, the activity of cephalosporins, vancomycin, daptomycin, linezolid, tobramycin and fusidic acid is significantly increased by reducing the amount of peptidoglycan synthesis and eDNA content of biofilms [103]. Although disrupting QS systems provides an effective way to lower biofilm recalcitrance, further research is needed to better understand the overall role of QS in biofilm resistance and tolerance to antibiotics.

## 6. Alternative Strategies for Combating Antibiotic Resistance

**Figure 4:**
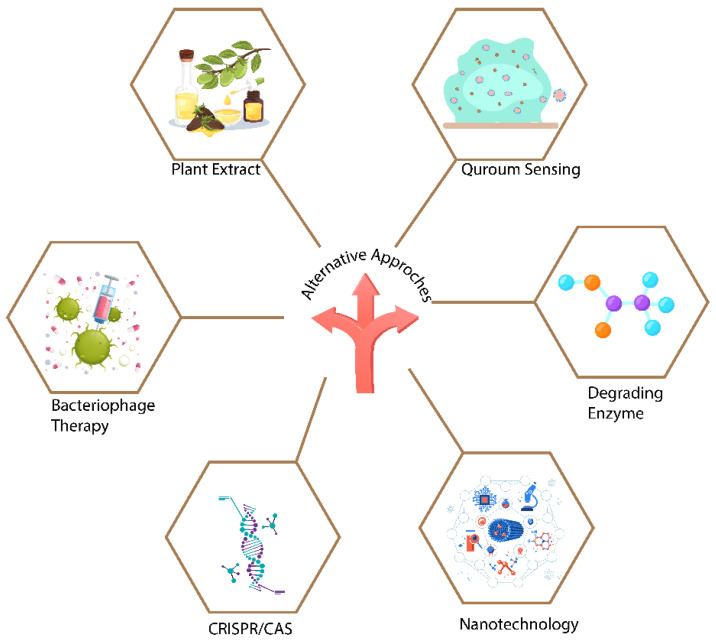
This figure represents new alternative options to antibiotics for combating antibiotic resistance in biofilm bacterial communities.

Biofilms present troubles for treatment through antibiotics by developing various resistance mechanisms, leading to severe medical complications in humans. As mentioned above, various classes of antibiotics are now ineffective for eradicating bacterial infections associated with biofilm formation. Therefore, new alternative options to antibiotics are required to combat antibiotic-resistant biofilm bacterial communities. In this part, some alternative strategies with the possible potential of eliminating biofilms or disrupting developed biofilms are discussed (Figure 4).

### 6.1. Phages Therapy

Phages are a group of rigorously host-specific viruses, which infect and require only bacteria for their survival and self-replication [104]. In recent years, slower new antibiotic discoveries and the rapid emergence of antibiotic resistance in bacteria have made phages a promising alternative therapy for eradicating antibiotic-resistant pathogenic bacteria [104]. So far, countless phages have been discovered [105] and reported with abilities to destroy bacteria, thus representing a potential candidate for preventing biofilm development [106]. Phages are even found to penetrate developed biofilms and disrupt biofilm structures with or without killing the resident bacteria [106]. Chan et al. [107] categorized phage-based removal of biofilms into three groups; first, intra- to extracellular breakdown of bacteria cells; second, extracellular to intracellular breakdown of bacteria cells; third, chemical-based dispersion of the biofilm matrix or components, e.g., EPS. Various phage-based treatments, e.g., phage therapy, phage-derived lysins, and phage-derived depolymerases, are used to degrade or disrupt the biofilm matrix or components [108]. Phage therapy has been proven to be an effective tool for exterminating biofilms developed by pathogenic bacteria.

In recent years, different studies have been carried out using phage therapy to combat bacterial biofilms, thus preventing biofilm-based antibiotic resistance [109,110,111]. In this regard, the first attempt was made using *Escherichia* virus T4 to eradicate developed biofilms of *E. coli* [112]. Researchers have also targeted eradicating biofilms developed on the surfaces of medical devices, e.g., prostheses and catheters [110,113]. In another study, the anti-biofilm activity of phage was assessed against prosthesis-related infections caused by *S. aureus* [114]. The results revealed a 3.3-fold reduction in biofilm biomass along with a decrease in the thickness and area of the biofilm. Similarly, Maszewska et al. [115] reported the phage potential involved in reductions and subsequent clearance of catheter-associated biofilms formed by *Proteus mirabilis*. In addition, phage therapy for eradicating biofilms developed by multi-drug-resistant bacteria has been assessed in recent years, e.g., *Enterobacter cloacae* [44], *P. aeruginosa* [116], and *S. gallinarum* [111] and *S. aureus* [114]. Furthermore, bacteriophage therapy can also be used for drug delivery to targeted areas through viral vectors [117]. These viral vectors of bacteriophage specifically target bacteria without affecting normal flora like antibiotics [118]. A study reported successful treatment of bacteriophage therapy against antibiotic-resistant *K. pneumoniae* in mice [119].

### 6.2. CRISPR/Cas Technique

The CRISPR/Cas (Clustered Regularly Interspaced Short Palindromic Repeats/CRISPR-associated genes) system is responsible for providing defense to bacteria against bacteriophages, which destroy bacteria [120]. However, recent research advances explored the CRISPR/Cas technique as an effective approach for combating antibiotic resistance. The CRISPR/Cas system can be used either for direct killing of pathogenic bacteria or to eradicate antibiotic resistance in bacteria. This technique presents high specificity and selectivity for attacking antibiotic resistance genes (ARGs) and eliminating bacteria in complex bacteria populations [121]. Direct killing of bacteria through CRISPR/Cas involves targeting genes on chromosomes and plasmids [122,123]. This mode, used for eliminating pathogenic bacteria, is demonstrated in *S. enterica* [124], *S. pneumonia* [125] and *S. aureus* [126]. Furthermore, *E. coli* was directly eliminated by targeting the *fucP* and *ogr* genes using the type I-E CRISPR/Cas system [124]. Similar target-killing effects were observed in *E. coli* using a CRISPR-Cas13a system [127]. In addition, Selle et al. [128] reported the potential of the type I-B CRISPR/Cas system for preventing and treating *Clostridioides difficile* infection by targeting bacterial chromosomal DNA [128].

The CRISPR/Cas system possesses the potential to restore bacterial susceptibility to antibiotics by neutralizing ARGs, thus eliminating pathogenic bacteria [120,126]. In this approach, ARGs are targeted, which are present on either the plasmid or chromosome and make drug-resistant bacteria sensitive to antibiotics. This technology has successfully suppressed targeted ARGs responsible for antibiotic-resistance pathogens [129]. A study reported that the CRISPR/Cas system made antibiotic-resistant *S. aureus* re-sensitive to kanamycin [125] and methicillin [130]. Rodrigues et al. [131] neutralized *tet(M)* and *erm(B)* genes responsible for tetracycline and erythromycin resistance in *E. faecalis*, respectively. The study showed a significant reduction in antibiotic resistance in *E. faecalis* in both in vitro and in vivo experiments. In another study, this tool was used to simultaneously remove drug-resistance genes on several plasmids [132]. Furthermore, the CRISPR/Cas system was also used for disrupting and neutralizing a wide range of ARGs in pathogenic bacteria, e.g., *tarH*, *tarO*, and *tarG* genes in *S. aureus* [133]; *sul2*, *blaOXA-55-like*, and *nmcR-like* drug resistance genes in *Shewanella algae* [134]; *tet(A)*, *ramR*, and *mgrB* genes in *K. pneumoniae* [135]; and the *mcr-1* gene in *E. coli* [99].

### 6.3. Nanoparticles Approach

Nanoparticles (NPs), ranging between 1 and 100 nm in size [136], are considered the most promising approach to deal with multidrug resistance and biofilm-based infections [137]. Biofilms provide shelter for bacterial communities by reducing antibiotic penetration, which is overcome by formulating NPs, which possess the ability to cross the biological barrier [138]. NPs have been reported to overcome current antibiotic resistance mechanisms, e.g., lowered uptake and higher efflux of drugs from microbial cells, biofilm development, and protective intracellular bacteria [137]. The existing treatment strategies for biofilm-based infections present several limitations that can be overcome by the nano-formulation of drugs. The main characteristic of such formulation is to cross biological barriers and reach target sites. A variety of NPs, e.g., metal NPs, green NPs and several other combinations of NPs, have been overserved to have antimicrobial and anti-biofilm properties [7,131]. In recent years, several reports confirmed the efficacy of NPs for the elimination of bacterial biofilm communities (Table 2) [7,131,132,137].

In the last few years, various types of NPs, e.g., nitric oxide-releasing nanoparticles (NO-NPs), chitosan-containing nanoparticles (chitosan-NPs) and metal-containing nanoparticles, have been designed and used against biofilms producing pathogenic bacteria [7,138,145,146]. Antibiotics like antimicrobial agents can be packaged within NPs for safe delivery to the target sites. For example, metal-based NPs utilize targeted drugs without any hindrance by the innate system of bacteria [147]. Metal-based NPs make therapeutic drugs reachable to the target without affecting the normal flora [148]. All of these NPs or NP-based drug deliveries work by using multiple mechanisms simultaneously, combating biofilm-producing bacteria, thus making antibiotic development unlikely. This makes NPs a promising approach to exterminating biofilm-based infections in humans [129].

Currently, various researchers are working on exploring nanoparticle potentials to eliminate bacterial biofilm communities and related threats of antibiotic resistance [149,150,151]. The CaF_2_-NPs were reported to suppress the genes associated with major virulence factors (*vicR*, *gtfC*, *ftf*, *spaP*, *comDE*) of *S. mutans* [150]. The suppressed genes were presumed to be involved in acid production, acid tolerance, cell adhesion, glucan synthesis, and QS, thus ultimately causing biofilm inhibition. In addition, Rajivgandhi et al. [152] reported an 80% reduction in biofilms produced by *K. pneumonia* using silver NPs (Ag NPs). The NPs were synthesized by using the marine seaweed *Gracilaria corticata*. In another study, copper oxide NPs (CuO NPs) were used against *K. pneumonia* and *Helicobacter pylori* biofilms [153]. The study showed that NPs inhibited biofilm formation by 92.5 and 99.5% for *K. pneumonia* and *H. pylori*, respectively. However, there are certain concerns associated with NPs while using them for medical applications. NPs must be precisely formulated to avoid influencing their functional properties and creating compatibility problems, which can disrupt desired outcomes during clinical applications. Therefore, all the parameters must be considered accurately while designing and formulating NPs to ensure efficacy against the biofilms and avoid any adverse effects, e.g., affecting body cells, creating immunological response, disposal and good diffusion into blood circulation and final discharge from the body via the kidney rout.

## 7. Concluding Remarks and Future Outlook

Bacterial communities develop biofilms by attaching to surfaces, which present properties different than planktonic cells, e.g., exhibiting a higher degree of resistance to antibiotics. Therefore, pathogens with biofilm-developing abilities are difficult to exterminate with antibiotic concentrations that would usually kill free-swimming planktonic cells. Based on their potential to withstand antibiotic treatments, pathogens demonstrate severe medical implications, e.g., chronic osteomyelitis, chronic otitis media, chronic prostatitis, colitis, conjunctivitis and otitis. The formation of biofilms is a well-regulated and organized developmental process in bacteria. Biofilm development is triggered by environmental factors, e.g., UV radiation, extreme temperatures, and exposure to antibiotics, and regulated by genetic players such as QS, c-di-GMP and sRNAs. The biofilm-producing bacterial communities present various mechanisms that contribute to antibiotic resistance and lead to the emergence of multidrug-resistant bugs. Bacteria provide different modes of action based on biofilms, e.g., the production of eDNA, antagonistic chemicals, and various mutations in gene regulators against different types of antibiotics. By keeping in view the medical problems and increased antibiotic resistance pattern in biofilms producing pathogenic bacteria, alternative treatment approaches must be considered as options in future research work, in addition to CRISPR/Cas, nanotechnology and phage therapy.

Firstly, various enzymes, e.g., Dispersin B (DspB), DNase I, and a-amylase, should be explored properly for their ability to degrade the biofilm matrix. This approach disrupts the structural components of biofilms, e.g., EPS, eDNA, and biofilm matrix, and would thus be helpful to enhance antibiotic penetration. In some previous studies, matrix-degrading enzymes were found to inhibit biofilm formation and degrade matured biofilms in pathogenic bacteria such as *P. aeruginosa*, *S. aureus* and *V. cholera* [154,155,156]. Secondly, plant-based treatments present a potential alternative option for combating biofilm-based diseases. Studies have shown the anti-biofilm potential of plant extracts. For example, extracts of *Polygonum cuspidatum* (Japanese knotweed), *Epimedium brevicornum* (rowdy lamb herb) and *Rhodiola crenulata* (arctic root) were found to be associated with inhibiting proprioni bacterium acne biofilm formation [157]. Finally, QS signaling genes can be disrupted by using a wide variety of inhibitors/compounds, thus presenting an alternative to eliminate biofilm-related infections. For example, halogenated furanone, acyclic diamine (ADM 3), usnic acid and ginseng have been observed to have great abilities to inhibit fungal and bacterial biofilm formation [39,158,159]. Sufficient research studies are required to find the exact extent of all these strategies for eliminating biofilms responsible for serious health complications.

## Figures and Tables

**Table 1:** Genes/clusters responsible for biofilm formation and biofilm-based antibiotic resistance in various bacteria.

S. No	Species	Gene/Clusters	Functions	References
1	*E. coli*	*HlyB–HlyD–TolC complex*	Exports hemolysin through biofilms, contributing to multi-drug resistance	[30]
		*RapA*	Responsible for biofilm-mediated resistance to penicillin	[31]
2	*P. aeruginosa*	*algACD*	Involved in alginate synthesis, an integral part of cystic fibrosis	[36]
		*ndvB*	Responsible for the expression of ethanol oxidation genes	[29]
		*tssC1*	Involved in biofilm-specific antibiotic resistance	[37]
3	*Salmonella typhimurium*	*csgD*	Responsible for biofilm formation	[38]
4	*S. aureus*	*icaABCD*	Enhancing virulence factors as well as for biofilm development and dispersion in methicillin resistance	[33]
5	*S. epidermidis*	*icaA* and *icaD*	Associated with the formation of slime and biofilm	[34]
6	*V. cholera*	*tssC1*	Virulent gene for toxin delivery in biofilm-related drug resistance	[32]

**Table 2:** Potentials of NPs against pathogenic bacterial biofilms.

S. No	Nanoparticles	NPs Size (nm)	Synthesis Method	Targeted Pathogens	References
1	Ag-NPs	2–10	Leaf extract of *Allophylus cobbe*	*P. aeruginosa*, *Shigella flexneri*, *S. aureus*, *S. pneumonia*	[139]
2	Au-NPs	10.2–11.5	Brust–Schiffrin two-phase synthesis method	*S. aureus*, *P. aeruginosa*	[140]
3	Fe_2_O_3_-NPs	11	Co-precipitation of ferric and ferrous ions	*Bacillus subtili*	[141]
4	MgO-NPs	50–70	Wet chemical method	*S. aureus*, *P. aeruginosa*	[142]
5	Ni-NPs	41.23	Solution reduction process	Against oral bacteria	[143]
6	ZnO-NPs	10–100	Co-precipitation method	*P. aeruginosa*, *Proteus vulgaris B. subtilis*, *B. pumilus*	[144]

## Data Availability

Data are contained within the article.
